# TNP-470 Suppresses the Tumorigenicity of HT1080 Fibrosarcoma Tumor Through the Inhibition of VEGF Secretion From the Tumor Cells

**DOI:** 10.1080/13577140120099182

**Published:** 2001-12

**Authors:** Mitsunori Kaya, Takuro Wada, Satoshi Nagoya, Satoshi Kawaguchi, Toshihiko Yamashita, Nobuyuki Yamamoto, Mitsunori Yoshimoto, Futoshi Okada, Seiichi Ishii

**Affiliations:** 1 Department of Orthopedic Surgery Sapporo Medical University School of Medicine S-1, W-16, Chuo-ku, Sapporo Hokkaido 060-8543 Japan; 2 Division of Cancer Pathobiology Research Section of Pathophysiology Institute for Genetic Medicine Hokkaido University, N-15, W-7, Kita-ku, Sapporo Hokkaido 060-0815 Japan

## Abstract

Angiogenesis inhibitors are a novel class of promising therapeutic agents for treating cancer. TNP-470, a systemic analogue of fumagillin, is an angiogenesis inhibitor capable of suppressing the tumorigenicity in several animal models even though the mechanisms of action have not been completely clarified. In the current study, we investigated the effects of TNP-470 on human fibrosarcoma cells *in vivo* and *in vitro*. The administration of TNP-470 could suppress the tumorigenicity of HT1080 fibrosarcoma tumor. The conditioned medium from HT1080 fibrosarcoma cells treated with TNP-470 inhibited the proliferation and migration of human endothelial cell line, HUVEC and ECV304. The concentration of VEGF in the conditioned medium from HT1080 cells treated with TNP-470 was lower than that of the cells without TNP-470 treatment, indicating that TNP-470 downregulates the secretion of VEGF from HT1080 cells. These findings strongly suggest that the direct action of TNP-470 on sarcoma cells inhibits angiogenesis through the downregulation of VEGF secretion and this angiogenesis suppression resulted in the inhibition of tumorigenicity of HT1080 fibrosarcoma tumo.


*Sarcoma* (2001) 5, 197–202

ORIGINAL ARTICLE

### TNP-470 suppresses the tumorigenicity of HT1080 fibrosarcoma tumor through the inhibition of VEGF secretion from the tumor cells

MITSUNORI KAYA[1], TAKURO WADA[1], SATOSHI NAGOYA[1], SATOSHI KAWAGUCHI[1], TOSHIHIKO YAMASHITA[1], NOBUYUKI YAMAMOTO[1], MITSUNORI YOSHIMOTO[1], FUTOSHI OKADA[2] AND SEIICHI ISHII[1]

[1] *Department of Orthopedic Surgery, Sapporo Medical University, School of Medicine, S-1, W-16, Chuo-ku, Sapporo, 060-8543, Hokkaido, Japan,* [2] *Division of Cancer Pathobiology, Research Section of Pathophysiology, Institute for Genetic Medicine, Hokkaido University, N-15, W-7, Kita-ku, Sapporo, 060-0815, Hokkaido, Japan*

**Summary**

Angiogenesis inhibitors are a novel class of promising therapeutic agents for treating cancer. TNP-470, a systemic analogue of fumagillin, is an angiogenesis inhibitor capable of suppressing the tumorigenicity in several animal models even though the mechanisms of action have not been completely clarified. In the current study, we investigated the effects of TNP-470 on human fibrosarcoma cells *in vivo* and *in vitro*. The administration of TNP-470 could suppress the tumorigenicity of HT1080 fibrosarcoma tumor. The conditioned medium from HT1080 fibrosarcoma cells treated with TNP-470 inhibited the proliferation and migration of human endothelial cell line, HUVEC and ECV304. The concentration of VEGF in the conditioned medium from HT1080 cells treated with TNP-470 was lower than that of the cells without TNP-470 treatment, indicating that TNP-470 downregulates the secretion of VEGF from HT1080 cells. These findings strongly suggest that the direct action of TNP-470 on sarcoma cells inhibits angiogenesis through the downregulation of VEGF secretion and this angiogenesis suppression resulted in the inhibition of tumorigenicity of HT1080 fibrosarcoma tumor.

### Introduction

Soft tissue sarcoma is a rare neoplasm which arise mainly in extremity or retroperitoneal space[1]. Despite progress in multimodarity treatment, the prognosis for the patients with soft tissue sarcoma is still poor.

It is well known that angiogenesis is essential for primary tumor growth, invasion and metastasis in several kinds of cancer. With regards to soft tissue sarcoma, tumor expression of vascular endothelial growth factor (VEGF), a potent angiogenic factor, was shown to correlate with malignant progression of disease[2,3]. The facts that tumor progression is associated with angiogenesis lead to the concept of angio-suppression as a new strategy for the treatment of cancer patients[4,7]. Recent study demonstrated that anti-angiogenic study using gene transfer of a cDNA coding for angiostatin[8], a potent angiogenic inhibitor, suppressed the mouse fibrosarcoma tumor growth *in vivo*[9]. This indicates that the growth of soft tissue sarcoma is, at least in part, angiogenesis dependent and anti-angiogenic therapy may be a novel approach for the treatment of the patients with soft tissue sarcoma.

The angiogenesis inhibitor TNP-470 (AGM-1470) is a synthetic analogue of fumagillin[10] but has stronger anti-angiogenic activity and fewer side effects than fumagillin[11]. Actually some clinical trials have been started for the treatment of cancer[12–14]. With regard to the effects of TNP-470 for bone and soft tissue sarcoma, several reports indicated that TNP-470 could suppress the tumorigenicity and the establishment of pulmonary metastasis in animal osteosarcoma[15,16].

In the current study, we examined whether TNP-470 could suppress the tumorigenicity of HT1080 fibrosarcoma tumor. Furthermore, to explore the interaction of TNP-470 with tumor cells, we have investigated whether the conditioned medium from the tumor cells treated with TNP-470 can inhibit the proliferation and motile activity of human endothelial cells. In addition, we have examined the secretion of several angiogenic factors from the tumor cells treated with TNP-470.

### Materials and methods

### *Cells and cell culture*

Human fibrosarcoma cell line HT1080, human umbilical vein endothelial cell (HUVEC) (Sanko Junyaku Co. Ltd., Tokyo, Japan) and spontaneous transformed human endothelial cell line ECV30[17]

Correspondence to: M. Kaya, Department of Orthopedic Surgery, Sapporo Medical University School of Medicine, S-1, W-16, Cho-ku, Sapporo, 060-8543, Hokkaido, Japan. Fax: 81-11-641-6026. E-mail: kaya@sap-cc.go.jp

1357–714X print/1369–1643 online/01/040197–06 © 2001 Taylor & Francis Ltd
DOI: 10.1080/13577140120099182

were maintained at 37°C at 5% $CO_2$ in Dulbecco's modified Eagle's medium (DMEM) with 10% fetal bovine serum (JRH Biosciences, Lenexa, KS) and penicillin/streptomycin.

*Materials*

TNP-470 was obtained from Takeda Chemical Industries Ltd. (Osaka, Japan). In *in vivo* experiments, TNP-470 was suspended in a vehicle composed of 0.5% ethanol plus 5% gum arabic in saline. In *in vitro* experiments, TNP-470 was dissolved in dimethylsulfoxide.

*Animals*

Female BALB/c nu/nu mice, which originated from the Central Institute for Experimental Animals (Kawasaki, Japan) were obtained from CLEA Japan Inc. (Tokyo, Japan). Mice 6 weeks old that weighed about 20g were used.

*Assay of HT1080 tumor growth*

HT1080 cells ($2\times10^6$/0.1 ml in phosphate-buffered saline) were s.c. injected into nude mice. TNP-470 (30 mg/kg) were given s.c. on alternate days for 4 weeks from day 1 after s.c. injection. The resulting tumors were measured with calipers and tumor volumes were estimated using the following formula: $V=a\times b^2/2$ (V, volume; a, the longest diameter; b, the shortest diameter).

*Proliferation assay of HT1080 cells in vitro*

HT1080 cells were plated on a 60 mm plastic dish on day 0 ($1\times10^6$) and cultured in DMEM supplemented with 10% FCS with or without 10 ng/ml of TNP-470. Cell growth was estimated on days 1 and 2 by counting the total number of cells on dishes. Each experiment was performed in triplicate.

*Preparation of conditioned medium*

First, $1\times10^6$ HT1080 cells were incubated with or without 10 ng/ml of TNP-470 for 24 hrs. The cells were washed twice with PBS and incubated with serum-free DMEM for 24 hrs. Condition medium was collected and kept at –80°C until use.

*Endothelial cell proliferation assay in vitro*

HUVEC or ECV304 cells were placed on a 60 mm plastic dish on day 0 ($1\times10^6$) and cultured in DMEM supplemented with 10% FCS. On day 1, the medium was replaced with condition medium from HT1080 cells treated with or without 10 ng/ml of TNP-470. Cell growth was estimated on days 2 and 3 by counting the total number of cells on dishes. Each experiment was performed in triplicate.

*In vitro motility assay*

Transwell culture chambers (Coaster Corp., Cambridge, USA) were used for the motility assay. HUVEC or ECV304 cells ($1\times10^5$) were suspended in condition medium from HT1080 cells treated with or without TNP-470 and added to the upper chamber. The lower chamber contained DMEM supplemented with 10% FCS and human cellular fibronectin (12.5 μg/ml, as a chemoatractant). Cells were incubated for 6 hrs. at 37°C in a $CO_2$ incubator. At the end of the incubation, cells on the upper surface of the filter were completely removed by wiping with a cotton swab. Cells were fixed with 5% glutaraldehyde and stained with Giemsa solution. Cells that moved to the lower surface of the filter were counted under a light microscope at a magnification of ×100. Each assay was done in triplicate.

*Matrigel plug assay for the evaluation of angiogenesis*

Mice in the tumor-removal and tumor-intact groups were injected s.c. with 0.5 ml of Matrigel (Becton Dickinson, Bedford, MA) containing 200 ng of recombinant basic fibroblast growth factor (Upstate Biotechnology, Lake Placid, NY). TNP-470 (30 mg/kg) was given s.c. on alternate days for 4 weeks. Matrigel pellets were harvested four weeks after the inoculation and reliquefied by incubation at 4°C overnight in 300 μl of PBS. Matrigel neovascularization was quantitatively determined by measuring the hemoglobin content of the liquefied pellets (Drabkin's method; Sigma Chemical Co. St Louis, MO). Five mice were used in each group.

*Angiogenic factors production*

Angiogenic factors, VEGF (vascular endothelial growth factor), bFGF (basic fibroblast growth factor) and PlGF (placenta growth factor), concentrations of conditioned medium from HT1080 cells ($1\times10^5$) treated with or without TNP-470 were measured by using an enzyme-linked immunosorbant assay kit (VEGF, IBL Fujioka, Japan; bFGF and PlGF, R & D Systems, Minneapolis, MN).

*Statistical analysis*

Tumor volume, cell proliferation, motile activity, and the serum levels of circulating factors were analyzed using the Student's *t* test. Statistical significance was defined as $p<0.05$.

### Results

*Effect of TNP-470 on the growth of HT1080 tumor in vivo and the proliferation of HT1080 cells in vitro*

The ability of TNP-470 to inhibit the neoplastic growth *in vivo* was evaluated in nude mice inoculated with HT1080 cells by s.c. injection. The effect of

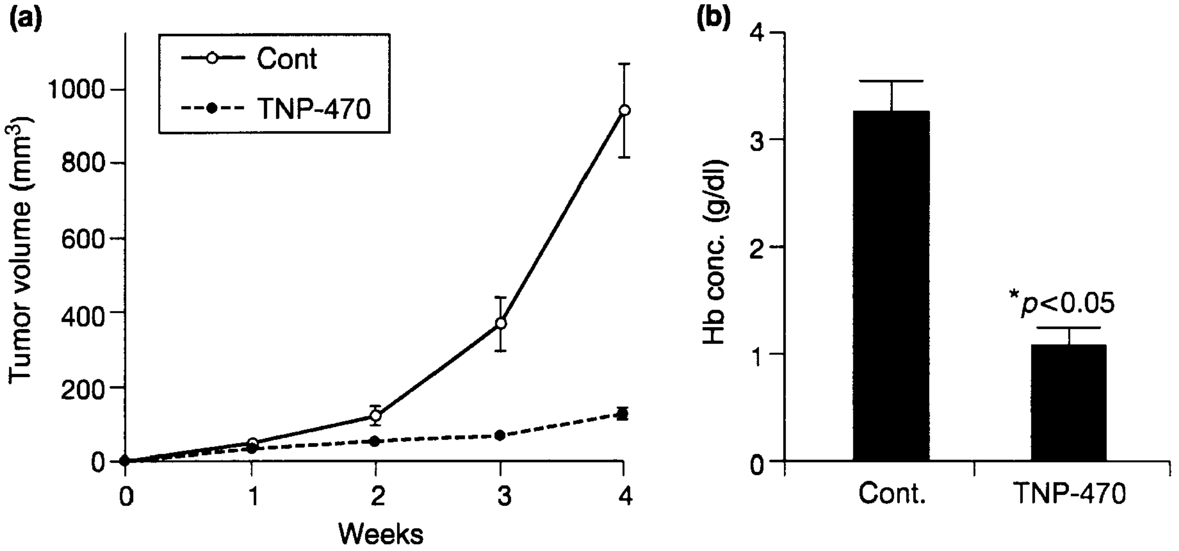

**Fig. 1.** *The effects of TNP-470 on the in vivo growth of HT1080 human fibrosarcoma cells. (a) HT1080 human fibrosarcoma cells ($1\times10^6$) were s.c. injected into nude mice. Tumor volume were measured weekly. Data are means from 3 independent experiments ± SEM. (b) Matrigel plug neovascularization was quantitated by measuring the hemoglobin content of the pellets using Drabkin's methods. Hemoglobin concentration was significantly lower in the mice of TNP-470 treated group. Columns, mean of 5 mice/group; bars, SE.*

TNP-470 at the concentration of 30 mg/kg was compared with the results of administration of a vehicle of 1% ethanol and 5% gum arabic in saline solution. As shown in Figure 1A, TNP-470 significantly inhibited the growth of HT1080 tumor ($p<0.05$). To examine whether TNP-470 can suppress the systemic angiogenic activity, Matrigel plug neovascularization assay was performed. The hemoglobin concentration of Matrigel plugs inoculated into the nude mice of the TNP-470 treated group was significantly lower than those of control group ($p<0.05$) indicating that the administration of TNP-470 could suppress the systemic angiogenic activity in nude mice (Figure 1B).

We next examined whether TNP-470 affected the proliferative activity of HT1080 cells *in vitro*. The addition of TNP-470, however, could not inhibit the proliferation of HT1080 cells (Figure 2). These findings indicate that TNP-470-mediated suppression of the tumorigenicity of HT1080 tumor was not due to the direct action of TNP-470 for the proliferation of HT1080 cells.

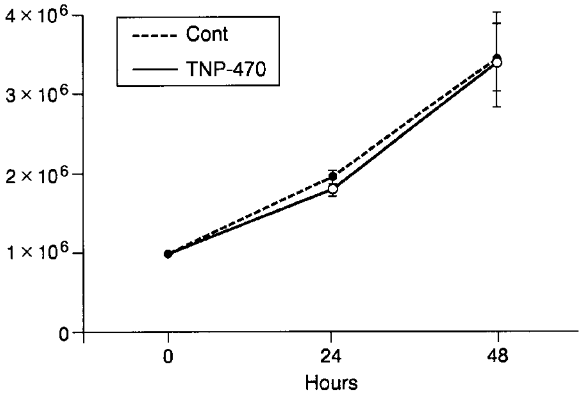

**Fig. 2.** *The effects of TNP-470 on the proliferation of HT1080 cells. HT1080 cells were incubated with TNP-470 (10 ng/ml) for 48 hrs. Cells were counted using hemocytometer. Data are means from 3 independent experiments ± SEM.*

### *Inhibitory effects of the conditioned medium from TNP-470-treated HT1080 cells on endothelial cell proliferation and migration*

To determine whether TNP-470 affects sarcoma cell-induced angiogenesis, we examined the effects of the conditioned medium from HT1080 cells treated with TNP-470 on the proliferation of HUVEC and ECV304 cells. In this experimental series, we prepared the conditioned medium as described in "Materials and Methods" in order to rule out the direct effects of TNP-470 on the endothelial cells. As shown in Figure 3, the addition of conditioned medium from HT1080 cells treated with TNP-470 to endothelial cells resulted in inhibition of endothelial cell proliferation. Furthermore, the addition of conditioned medium from HT1080 cells treated with TNP-470 inhibited the motile activity of endothelial cells (Figure 4).

### *Inhibitory effects of TNP-470 on VEGF production by HT1080 cell*

Angiogenic factors are potent mitogen for endothelial cells[18]. Among angiogenic factors, VEGF, bFGF and PlGF are considered to be most commonly secreted from the tumor cells. Because the conditioned medium from HT1080 cells treated with TNP-470 inhibited the proliferation of

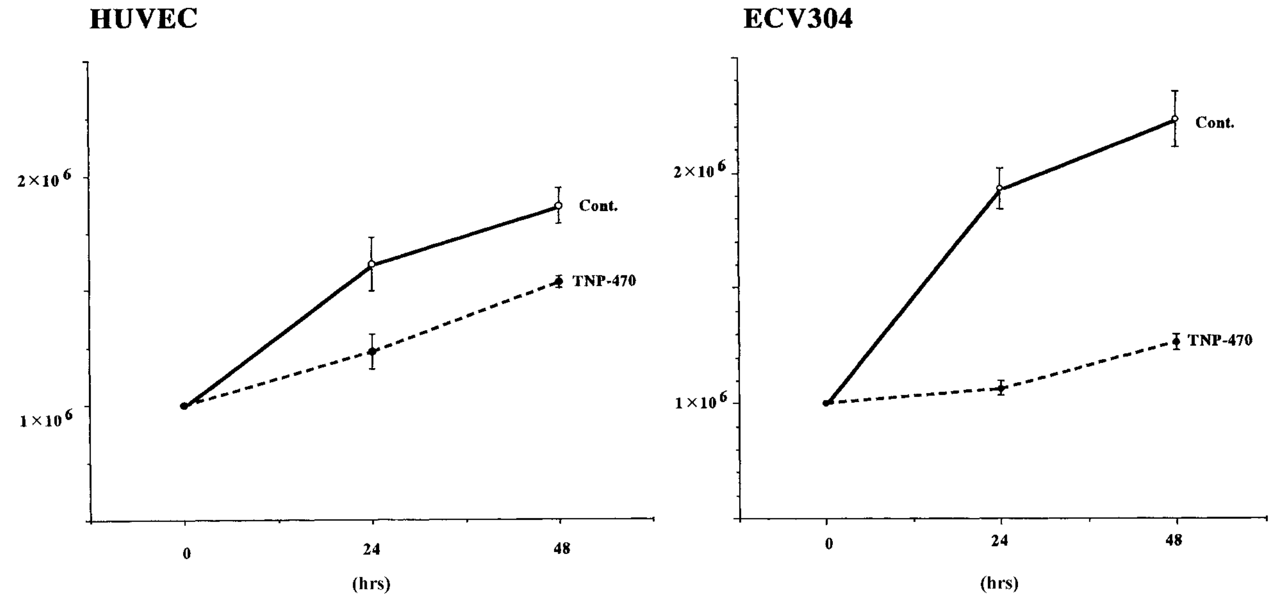

**Fig. 3.** *The effects of conditioned medium from HT1080 treated with TNP-470 on endothelial proliferation. HUVEC and ECV304 were incubated with the conditioned medium from HT1080 treated with or without TNP-470 (10 ng/ml) for 48 hrs. Cells were counted using hemocytometer. Data are means from 3 independent experiments ± SEM.*

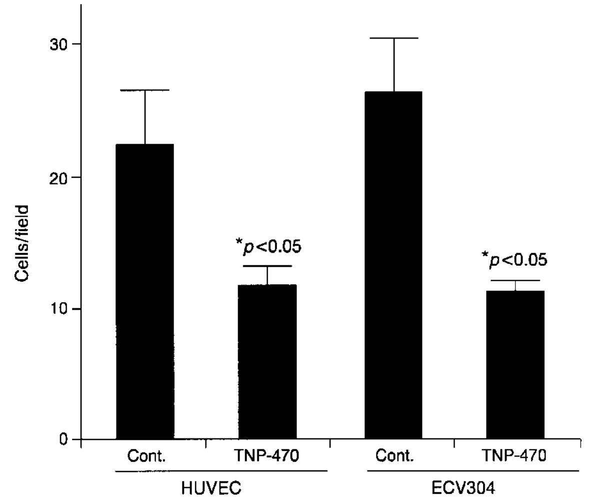

**Fig. 4.** *The effects of conditioned medium from HT1080 treated with TNP-470 on endothelial migration. Motility of HUVEC and ECV304 cells suspended in the conditioned medium from HT1080 treated with or without TNP-470 (10 ng/ml) was determined by chemotaxis assays using a transwell cell culture chamber. Data are means from 3 independent experiments ± SEM.*

HUVEC and ECV304 cells, we assessed whether the direct action of TNP-470 on HT1080 cells could suppress the secretion of angiogenic factors from the sarcoma cells. As shown in Figure 5, the concentration of VEGF in cultures of HT1080 cells treated with TNP-470 was significantly lower than that of control cells ($p<0.05$). The treatment of TNP-470 had no effects on the secretion of bFGF and PlGF. These results indicate that anti-angiogenic activity of TNP-470 is mediated, at least in part, the suppression of VEGF secretion from the tumor cells.

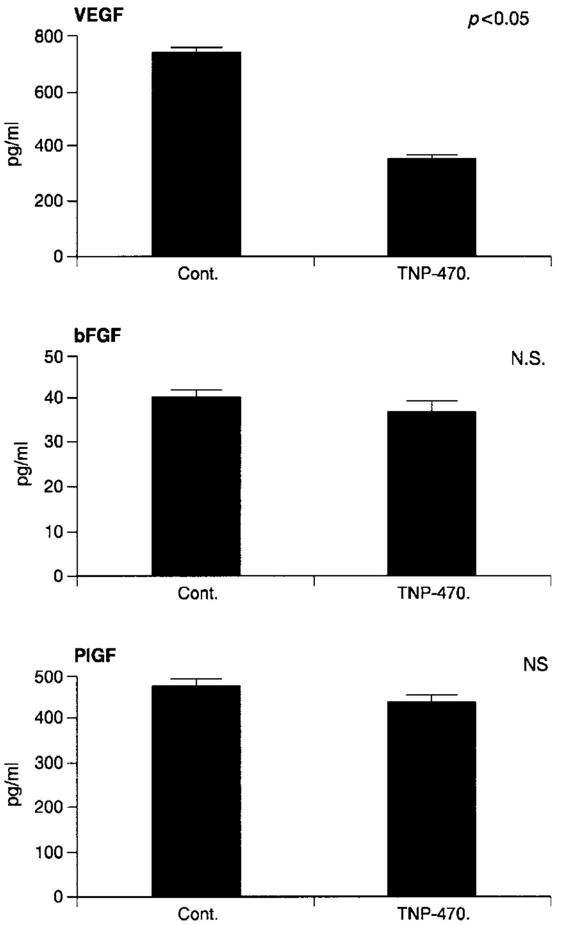

**Fig. 5.** *The effects of TNP-470 on the angiogenic factor production by HT1080 cells. HT1080 human fibrosarcoma cells ($1\times10^5$) were cultured for 24 hrs. with or without TNP-470 (10 ng/ml). The concentration of VEGF, bFGF and PlGF which were secreted into the culture media were assayed with enzyme immunoassay kits. Data are means from 3 independent experiments ± SEM.*

### *Inhibitory effects of TNP-470 on tumor-mediated angiogenesis*

We finally examined whether angiogenesis-inducing ability of the conditioned medium from HT1080 cells treated with TNP-470 was suppressed using a Matrigel plug neovascularization assay. In brief, Mice were injected s.c. with 0.5 ml of Matrigel containing 50 μl of the conditioned medium from HT1080 cells treated with or without TNP-470. Matrigel neovascularization was quantitatively determined by measuring the hemoglobin content of the liquefied pellets. The hemoglobin concentration of Matrigel plugs with the conditioned medium from HT1080 cells treated with TNP-470 was significantly lower than those from control cells ($p<0.05$) (Figure 6).

### Discussion

In the present study, we have demonstrated that 1) TNP-470 can inhibit the tumorigenicity of HT1080 fibrosarcoma cells, 2) conditioned medium from HT1080 cells treated with TNP-470 can inhibit the proliferation and motile activity of human endothelial cells, and 3) TNP-470 can downregulate the secretion of VEGF from HT1080 cells. These results indicate that the downregulation of VEGF secretion from tumor cells by TNP-470 caused the suppression of tumor neoangiogenesis which lead to the inhibition of HT1080 tumor growth.

With regard to the mechanisms of antiangiogenic activity of TNP-470, several reports indicated that TNP-470 could act on endothelial cells directly and inhibit neovascularization by preventing its proliferation[19–24]. On the other hand, it has been also reported that TNP-470 could act on the tumor cells directly and downregulated the production of angiogenic factors by tumor cells, thereby impedes tumoral angiogenesis[25–27].

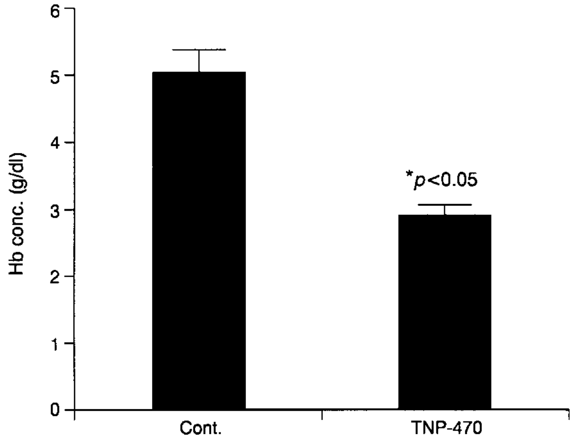

**Fig. 6.** *Angiogenic ability of the conditioned medium from TNP-470 treated HT1080 cells. Matrigel plug neovascularization was quantitated by measuring the hemoglobin content of the pellets with the conditioned medium from HT1080 cells treated with or without TNP-470 using Drabkin's methods. Hemoglobin concentration of the pellets with the conditioned medium from HT1080 cells treated with TNP-470 was significantly lower. Columns, mean of 5 mice/group; bars, SE.*

Recent study demonstrated that the effects of TNP-470 was more remarkable on angiogenic factor-positive tumors than on angiogenic factor-negative tumors. And this suppressive effect was accompanied by the downregulation of the angiogenic factor production by tumor cells suggesting the possibility that the direct action of TNP-470 on tumor cells is more significant than that on the endothelial cells[27].

Vascular endothelial growth factor (VEGF) is a homodimeric protein recently identified as a mitogen for endothelial cells *in vitro*, and an angiogenesis-promoting factor *in vivo*[28–31]. The data we have shown here indicates that TNP-470 could suppress the secretion of VEGF from HT1080 cells resulting in the inhibition of endothelial cell proliferation and motility. Thus, the inhibitory effects of TNP-470 for the tumorigenicity of HT1080 tumors considered to be, at least in part, due to the suppressed secretion of VEGF from the tumor cells. Furthermore, the secretion of other angiogenic factors, bFGF and PlGF, have not been downregulated after the treatment of TNP-470 indicating that VEGF is one of the potent angiogenic factors which are affected by the treatment of TNP-470.

We have recently demonstrated that the expression of VEGF in primary osteosarcoma is significantly correlated with the development of pulmonary metastasis and poor prognosis[32]. These findings suggest the possibility that VEGF-mediated angiogenesis may play a critical role in the progression of sarcoma. Several reports have also demonstrated that TNP-470 successfully suppressed the tumorigenicity and development of pulmonary metastasis in experimental animal sarcoma models[15,16]. Now, with the current data which we have demonstrated here, it is conceivable that anti-angiogenic drugs like TNP-470 could be indicated for the treatment of VEGF positive sarcoma.

In conclusion, the present study provides evidence for the molecular mechanism of the anti-angiogenic activity of TNP-470 and also the possibility that anti-angiogenic drugs could be employed for the treatment of soft tissue sarcoma, targeting angiogenesis as a new therapeutic strategy.

### Acknowledgements

We thank M. Ono, M. Naka for their excellent secretarial assistance, Dr. M. Hosokawa for helpful comments and M. K. Barrymore for comments on the manuscript. This work was supported in Grant-in-Aid 11307026 from the Ministry of Health and Welfare of Japan.

### References

1. Billingsly KB, Burt ME, Jara E, Ginsberg RJ, Woodruff JM, Leung DHY, Brennan MF. Pulmonary metastases from soft tissue sarcoma: analysis of patterns of diseases and postmetastasis survival. *Ann Surg* 1999; 5: 602–612.
2. Yudoh K, Kanamori M, Ohmori K, Yasuda T, Aoki M, Kimura T. Concentration of vascular endothelial growth factor in the tumour tissue as a prognostic factor of soft tissue sarcomas. *Br J Cancer* 2001; 84: 1610–5.
3. Chao C, Al-Saleem T, Brooks JJ, Rogatko A, Kraybill WG, Eisenberg B. Vascular endothelial growth factor and soft tissue sarcomas: tumor expression correlates with grade. *Ann Surg Oncol* 2001; 8: 260–7.
4. Folkman J. Tumor angiogenesis: therapeutic implications. *N Engl J Med* 1971; 285: 1182–1186.
5. Folkman J. Anti-angiogenesis: new concept for therapy of solid tumors. *Ann Surg* 1972; 175: 4–6.
6. Folkman J. What is the evidence that tumors are angiogenesis dependent? *J Natl Cancer Inst* 1990; 82: 4–6.
7. Folkman J. Angiogenesis in cancer, vascular, rheumatoid and other disease. *Nature Med* 1995; 1: 27–31.
8. O'Reilly MS, Holmgren L, Shing Y, Chen C, Rosenthal RA, Moses M, Lane WS, Cao YE, Sage H, Folkman J. Angiostatin: a novel angiogenesis inhibitor that mediates the suppression of metastases by a Lewis lung carcinoma. *Cell* 1994; 79: 315–328.
9. Cao Y, O'Reilly MS, Marshall B, Flynn E, Ji RW, ans Folkman J. Expression of angiostatin cDNA in a murine fibrosarcoma suppresses primary tumor growth and produces long-term dormancy of metastases. *J Clin Invest* 1998; 5: 1055–1063.
10. Ingber D, Fujita T, Kishimoto S, Sudo K, Kanamaru T, Brem H, Folkman J. Synthetic analogues of fumagillin that inhibit angiogenesis and suppress tumor growth. *Nature* 1990; 348: 555–557.
11. Kusaka M, Sudo K, Fujita T, Marui S, Itoh F, Ingber D, Folkman J. Potent anti-angiogenic action of AGM-1470: comparison to the fumagillin parent. *Biochem Biophys Res Commu* 1991; 174: 1070–1076.
12. Bahargava P, Marshall JL, Rizvi N, Dahut W, Yoe J, Figuera M, Phipps K, Ong VS, Kato A, Hawkins MJ. A phase I and pharmacokinetic study of TNP-470 administered weekly to patients with advanced cancer. *Clin Cancer Res* 1999; 5: 1989–1995.
13. Kudelka AP, Levy T, Verschraegen CF, Edwards CL, Piamsomboon S, Termrungruanglert W, Freedman RS, Kaplan AL, Kieback DG, Meyers CA, Jaeckle KA, Loyer E, Steger M, Mante R, Mavligit G, Killian A, Tang RA, Gutterman JU, Kavanagh JJ. A phase I study of TNP-470 administered to patients with advanced squamous cell cancer of the cervix. *Clin Cancer Res* 1997; 3: 1501–1505.
14. Stadler WM, Kuzel T, Shapiro C, Sosman J, Clark J, Vogelzang NJ. J Multi-institutional study of the angiogenesis inhibitor TNP-470 in metastatic renal carcinoma. *Clin Oncol* 1999; 17: 2541–2545.
15. Morishita T, Mii Y, Miyauchi Y, Miura S, Honoki K, Aoki M, Kido A, Tamai S, Tsutsumi M, Konishi Y. Efficacy of the angiogenesis inhibitor O-(chloroacetyl-carbamoyl)fumagillol (AGM-1470) on osteosarcoma growth and lung metastasis in rats. *Jpn J Clin Oncol* 1995; 25: 25–31.
16. Mori S, Ueda T, Kuratsu S, Hosono N, Izawa K, Uchida A. Suppression of pulmonary metastasis by angiogenesis inhibitor TNP-470 in murine osteosarcoma. *Int J Cancer* 1995; 29: 148–152.
17. Takahashi K, Sawasaki Y, Hata J, Mukai K, Goto T. Spontaneous transformation and immortalization of human endothelial cells. *In Vitro Cell Dev Biol* 1990; 26: 265–274.
18. Relf M, LeJeune S, Scott PA, Fox S, Smith K, Leek R, Moghaddam A, Whitehouse R, Bicknell R, Harris AL. Expression of the angiogenic factors vascular endothelial cell growth factor, acidic and basic fibroblast growth factor, tumor growth factor beta-1, platelet-derived endothelial cell growth factor, placenta growth factor, and pleiotrophin in human primary breast cancer and its relation to angiogenesis. *Cancer Res* 1997; 57: 963–969.
19. Wassberg E, Hedborg F, Skoldenberg E, Stridsberg M, Christofferson R. Inhibition of angiogenesis induces chromaffin differentiation and apoptosis in neuroblastoma. *Am J Pathol* 1999; 154: 395–403.
20. Ohta S, Nishio K, Kubota N, Ohmori T, Funayama Y, Ohira T, Nakajima H, Adachi M, Saijo N. Characterization of a taxol-resistant human small-cell lung cancer cell line. *Jpn J Cancer Res* 1994; 85: 290–297.
21. Lu Q, Luduena RF. Removal of beta III isotype enhances taxol induced microtubule assembly. *Cell Struct Funct* 1993; 18: 173–182.
22. Antoine N, Greimers R, De Roanne C, Kusaka M, Heinen E, Simar LJ, Castronovo V. AGM-1470, a potent angiogenesis inhibitor, prevents the entry of normal but not transformed endothelial cells into the G1 phase of the cell cycle. *Cancer Res* 1994; 54: 2073–2076.
23. Kusaka M, Sudo K, Matsutani E, Kozai Y, Marui S, Fujita T, Ingber D, Folkman J. (1994). Cystostatic inhibition of endothelial cell growth by the angiogenesis inhibitor TNP-470 (AGM-1470). *Br J Cancer* 69: 212–216.
24. Maier JA, Delia D, Thorpe PE, Gasparini G. In vitro inhibition of endothelial cell growth by the antiangiogenic drug AGM-1470 (TNP-470) and the anti-endoglin antibody TEC-11. *Anticancer Drugs* 1997; 8: 238–244.
25. Shishido T, Yasoshima T, Denno R, Mukaiya M, Sato N, Hirata K. Inhibition of liver metastasis of human pancreatic carcinoma by angiogenesis inhibitor TNP-470 in combination with cisplatin. *Jpn J Cancer Res* 1998; 89: 963–969.
26. Emoto M, Ishiguro M, Iwasaki H, Kikuchi M, Kawarabayashi T. TNP-470 inhibits growth and the production of vascular endothelial growth factor of uterine carcinosarcoma cells in vitro. *Anticancer Res* 2000; 20: 601–604.
27. Mori J, Haisa M, Naomoto Y, Takaoka M, Kimura M, Yamatsuji T, Notohara K, Tanaka N. Suppression of tumor growth and downregulation of platelet-derived endothelial cellgrowth factor/thymidine phosphorylase in tumor cells by angiogenesis inhibitor TNP-470. *Jpn J Cancer Res* 2000; 91: 643–650.
28. Gospodarowics D, Abraham JA, Schilling J. Isolation and characterization of a vascular endothelial cell mitogen produced by pituitary-derived follicles stellate cells. *Proc Natl Acad Sci USA* 1989; 86: 7311–7315.
29. Ferrara N, Nenzel WJ. Pituitary follicular cells secrete a novel heparin-binding growth factor specific for endothelial cells. *Biochem Biophys Res Commun* 1989; 161: 851–858.
30. De Vries C, Escobedo JA, Ueno H, Houck K, Ferrara N, Williams LT. The fms-like tyrosine kinase, a receptor for vascular endothelial growth factor. *Science* 1992; 255: 989–991.
31. Shibuya M, Yamaguchi S, Yamane A, Ikeda T, Tojo A, Matsushime H, Sato T. Nucleotide sequence and expression of a novel human receptor type tyrosine kinase gene (flk) closely related to the fms family. *Oncogene* 1990; 5: 519–524.
32. Kaya M, Wada T, Akasuka T, Kawaguchi S, Nagoya S, Shindoh M, Higashino F, Mezawa F, Okada F, Ishii S. Vascular endothelial growth factor (VEGF) expression in untreated osteosarcoma is predictive of pulmonary metastasis and poor prognosis. *Clinical Cancer Res* 2000; 6: 572–577.
